# The Role of FGL2 in the Pathogenesis and Treatment of Hepatitis C Virus Infection

**DOI:** 10.5041/RMMJ.10004

**Published:** 2010-07-02

**Authors:** Itay Shalev, Nazia Selzner, Ahmed Helmy, Katharina Foerster, Oyedele A. Adeyi, David R. Grant, Gary Levy

**Affiliations:** Multi Organ Transplant Program, University Health Network, University of Toronto, Toronto, Canada

**Keywords:** FGL2, Treg, immunity, regulation, HCV, MHV-3

## Abstract

Chronic hepatitis C virus (HCV) infection is a leading cause of liver disease worldwide and remains the most common indication for liver transplantation. The current standard of care leads to a sustained viral response of roughly 50% of treated patients at best. Furthermore, anti-viral therapy is expensive, prolonged, and associated with serious side-effects. Evidence suggests that a poor response to treatment may be the result of a suppressed anti-viral immunity due to the presence of increased numbers and activity of CD4^+^CD25^+^Foxp3^+^ regulatory T cells (Treg cells). We and others have recently identified fibrinogen-like protein 2 (FGL2) as a putative effector of Treg cells, which accounts for their suppressive function through binding to Fc gamma receptors (FcγR). In an experimental model of fulminant viral hepatitis, our laboratory showed that increased plasma levels of FGL2 pre- and post-viral infection were predictive of susceptibility and severity of disease. Moreover, treatment with antibody to FGL2 fully protected susceptible animals from the lethality of the virus, and adoptive transfer of wild-type Treg cells into resistant *fgl2-*deficient animals accelerated their mortality post-infection. In patients with HCV infection, plasma levels of FGL2 and expression of FGL2 in the liver correlated with the course and severity of the disease. Collectively, these studies suggest that FGL2 may be used as a biomarker to predict disease progression in HCV patients and be a logical target for the development of novel therapeutic approaches for the treatment of patients with HCV infection.

## INTRODUCTION

Hepatitis C virus (HCV) infection is a leading cause of liver disease with an estimated 200 million people infected worldwide.^1^ If untreated, the inflammatory response to the virus promotes hepatic fibrosis and development of cirrhosis which may be complicated by hepatocellular cancer (HCC). As a result, HCV infection has now become the most common indication for liver transplantation. Unfortunately, HCV reinfection of the graft occurs universally and is associated with an aggressive course in a proportion of patients, leading to graft cirrhosis in 10%–30% of recipients within 3–5 years.^2^ Therefore, the 5-year survival of HCV-positive liver transplant recipients is overall significantly lower than that of HCV-negative patients.^2^

The goal of HCV treatment is to prevent hepatic (cirrhosis and hepatocellular cancer) and extrahepatic complications by permanently eradicating the virus. At present, the standard of care for treating chronic HCV is the combination of weekly subcutaneous injections of pegylated interferon-α (PegIFNα) and ribavirin (RBV) for 24–48 weeks, depending on the viral genotype. Treatment with PegIFNα/RBV needs to be prolonged (6–12 months), and compliance is a necessity. To add to this, the treatment is expensive and is associated with significant side-effects.^3^ The rate of a sustained viral response (SVR) following this therapy is at best 50% overall.^4^–^6^

Presently, a number of host and viral factors are associated with response to therapy. These include race, viral genotype, alcohol intake, and liver histology (amount of steatosis and stage of fibrosis).^7^–^10^ Genetic diversity of the host contributes to the outcome of HCV infection and antiviral treatment. The sequencing of the human genome together with the development of new technologies, such as gene expression profiling and high-throughput protein analysis, has provided opportunities for rapid and accurate characterization of gene expression in tissues, and for the detection of individual host genetic polymorphisms. For example, our group has recently identified consistent patterns of gene expression in the pre-treatment liver biopsies which were predictive of treatment response.^11^ Identification of biomarkers to predict anti-viral treatment response would provide important diagnostic reagents in the management of HCV and may allow for the development of novel therapeutics for patients with HCV infection.

## IMMUNITY TO HCV

Both the innate and adaptive immune responses are important for viral clearance.^12^ In innate immunity, a number of innate effector cells and cytokines have been shown to be important for clearance of HCV infection. Natural killer (NK) cells play a key role in the innate anti-viral immune responses to HCV.^12^ Additionally, production of type 1 interferons (IFN) is important in first-line defense against HCV infection. The non-structural protein 3 (NS3)/4A protease and NS5A of HCV have been shown to impair both IFN production and IFN responsiveness, which would contribute to the inability to mount effective immune responses to HCV.^9^

In adaptive immunity, robust CD4^+^ and CD8^+^ T cell responses are associated with clearance of HCV.^13^ Impaired CD4^+^ and CD8^+^ T cell responses are known to be associated with chronic HCV. Patients who have spontaneously recovered from HCV infection maintain virus-specific CD4^+^ and CD8^+^ T cell responses that are readily detectable in their blood.^13^–^15^ These responses contribute to control and/or clearance of HCV as shown in a non-human primate model of HCV infection. In this model, depletion of either CD4^+^ or CD8^+^ T cells prior to challenge with HCV leads to chronic infection with high viral titers.^16^

Patients with chronic HCV typically display narrowly focused and weak HCV-specific T cell responses.^17^,^18^ Virus-specific T cells isolated from the peripheral blood of these patients appear to have lost most of their ability to proliferate and to produce cytokines (interleukin (IL)-2 and IFN-γ). In addition, CD8^+^ T cells display reduced cytotoxicity. In the absence of pre-existing defects in adaptive immunity, such as immunosuppression associated with malnutrition, human immunodeficiency virus (HIV) co-infection, or renal failure, this CD8^+^ T cell dysfunction has been attributed to high levels of persisting viral antigens.

An additional factor that influences the functional capacity of the CD8^+^ T cell pool is activation and stimulation by CD4^+^ T helper cells. CD4^+^ T cells are involved either by directly activating dendritic cells (DC) and CD8^+^ T cells via CD40-dependent co-stimulation or by indirectly supporting B cell and CD8^+^ T cell responses by secretion of cytokines, such as IL-4 and IL-2. In the mouse model of lymphocytic choriomeningitis virus (LCMV)-induced hepatitis, CD8^+^ T cell function was dependent on CD4^+^ T helper cell responses.^19^ That was shown by the observation that CD8^+^ T cell function was reduced in the absence of CD4^+^ T cells.^19^ Moreover, as shown in the non-human primate model of HCV infection, protective CD8^+^ T cell immunity may require CD4^+^ T helper cells not only in the primary infection but also after recovery, at the time of re-challenge.^20^

Treatment of acute infection with PegIFN results in high rates of virus clearance, in part by an efficient early stimulation of anti-HCV CD4^+^ Th1 responses.^21^,^22^ It has been recently demonstrated that chronic HCV-infected patients with mild or absent disease had circulating memory CD4^+^ T cells that recognized NS3 and HCV core antigens in contrast to those with severe disease.^23^ Similarly, chronic HCV patients who responded to treatment with IFN also demonstrate an increased Th1 cytokine profile and persistent viral-specific CD4^+^ responses, responses which are weak or absent in non-responders.^24^

## ROLE OF TREG CELLS IN THE PATHOGENESIS OF HCV INFECTION

Foxp3^+^CD4^+^CD25^+^ regulatory T cells (Treg cells), which constitute 5%–10% of peripheral CD4^+^ T cells in humans, are known to be actively engaged in the negative control of physiological and pathological immune responses.^25^

Treg cells have a broad T cell receptor repertoire that can recognize various self and non-self antigens. It has been suggested that the immune system employs Treg cells to maintain self-tolerance by suppressing the activation and expansion of self-reactive lymphocytes that might otherwise cause autoimmune disease.^25^ A controlled balance between initiation and down-regulation of the host immune response is vital in maintenance of immune homeostasis. A number of studies have suggested that depletion or reduction of Treg cells leads to enhanced immune responses against various infectious pathogens including HCV.^26^,^27^ A higher proportion of Treg cells was found in patients with chronic HCV infection when compared with successfully treated and/or healthy controls.^26^,^28^–^30^*In vitro* depletion of these cells results in increased HCV-specific T cell responsiveness.^28^,^29^ Thus, Treg cells appear to suppress the effector response of virus-specific T cells in patients with chronic HCV infection.

Treg cells have been shown to exert their suppressive activity through a number of different pathways. Production of immunoregulatory cytokines has been proposed as a major mechanism by which Treg cells mediate their function. Treg cell suppressive cytokines that have been described in the literature include transforming growth factor (TGF)-β, IL-10, and IL-35.^31^ These molecules have been shown to play a key role in the suppressive activity of Treg cells.^31^,^32^ Recently, we and others have identified the fibrinogen-like protein 2 (*fgl2*) as a putative effector gene of Treg cells and other regulatory T cell subsets, including CD8^+^CD45RC^low^ T cells, CD8αα^+^ T cells in the intestine, and CD4^−^CD8^−^ double negative (DN) T cells.^31^,^33^–^36^

## FIBRINOGEN-LIKE PROTEIN 2 (FGL2)

FGL2, also known as fibroleukin, was first cloned from cytotoxic T lymphocytes and was classified as a member of the fibrinogen superfamily due to its homology (36%) with fibrinogen β and γ chains.^37^ The *fgl2* gene, which has been localized to chromosome 7 and 5 in humans and mice, respectively, is composed of two exons that are separated by one intron. The *fgl2* promoter contains *cis* element consensus sequences for the binding of various transcription factors, including Ets, AP1, Sp1, TCF1, Ikaros, and CEBP.^38^

The *fgl2* gene encodes a protein of 432 amino acids in mice and 439 amino acids in humans. The deduced protein sequence contains a predicted signal peptide, five N-linked glycosylation sites, and conserved cysteine residues. Under non-reducing conditions the molecular mass of the protein is 250–300 kDa, and in reducing condition it is 64–70 kDa, indicating that FGL2 in its natural state forms a tetrameric complex.^39^,^40^ Based on sequence and structural analysis, it is predicted that the encoded protein is composed of two major regions, the N-terminal domain and the carboxyl-terminus. The N-terminal domain is proposed to have a linear conformation due to the presence of α-helical region and several conserved cysteine residues, which can promote coiled-coil formation. The 229-amino-acid-long carboxyl-terminus consists of a highly conserved globular domain, known as the fibrinogen-related domain (FRED), that is characteristic of the fibrinogen-related protein superfamily. The overall identity between the mouse and human FGL2 is 78%, but within the FRED domain the two proteins share 90% homology.^41^

**Figure 1.**
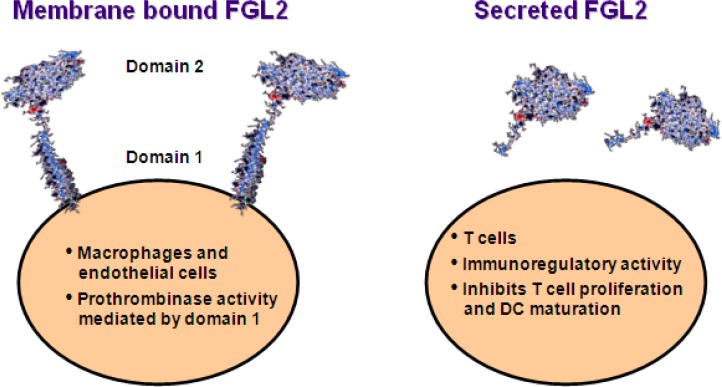
Schematic view of the two forms of FGL2 with their respective function as was previously reported. Macrophages and endothelial cells express a membrane-associated FGL2, which acts as a prothrombinase, while T cells produce a secreted form of the protein that plays a role in regulation of immune responses. DC, dendritic cells.

In macrophages and endothelial cells, FGL2 is primarily expressed as a membrane-associated protein (Figure 1), which has prothrombinase activity with the ability to generate thrombin directly from prothrombin.^42^ By a combination of site-directed mutagenesis and production of truncated proteins, it was shown that the serine 89 residue of the N-terminal domain is critical for the prothrombinase activity of FGL2.^42^ FGL2 prothrombinase activity has been implicated in the pathogenesis of various human and experimental models of disease including viral hepatitis, xeno- and allotransplantation rejection, and fetal loss syndrome.^43^–^46^

FGL2 is secreted by regulatory T cells (Figure 1), inhibits DC maturation and function, and induces B cell apoptosis.^35^,^36^,^40^,^47^ The C-terminal globular portion of FGL2 has been suggested to account for the immunomodulatory function of the molecule.^47^ FGL2 exerts its regulatory activity by binding to Fc gamma receptors (FcγR) that are expressed differentially on antigen-presenting cells including monocyte/ macrophages, DC, B cells, and endothelial cells.^39^ The regulatory activity of FGL2 has been implicated in inhibition of allograft rejection^39^ and autoimmunity,^35^ and the pathogenesis of experimental and human viral infections, including in patients with HIV, severe acute respiratory syndrome (SARS), and hepatitis B virus.^36^,^45^,^48^,^49^

## FGL2 AS A REGULATOR OF IMMUNE RESPONSES

Although the prothrombinase activity of the membrane-associated FGL2 expressed by macrophages and endothelial cells has been well established by many studies, the exact role of T cell-secreted FGL2 remains undefined. Recent studies by our group and other laboratories have suggested that FGL2 might be important in the regulation of adaptive immune responses. This would be consistent with the observation that other members of the fibrinogen-related superfamily that contain the FRED domain have previously been shown to have immunoregulatory activity in addition to their role in coagulation.^47^ Other members of the fibrinogen-related superfamily include tenascin that can inhibit T cell activation in response to alloantigens and Con A, a fibrinogen that has the ability to trigger a series of intracellular signaling events and cellular responses upon interaction with integrins and angi-opoietins, and ficolins, which have also been shown to have the ability to regulate host immune responses.^47^

Clinical studies have also suggested a role for FGL2 in immunoregulation. Kohno et al. showed that expression of *fgl2* is down-regulated in patients with both acute and chronic adult T cell leukemia/lymphoma.^50^ Patients with acute and chronic hepatitis B infection have been reported to express high levels of FGL2 in their livers.^45^ In addition, genomic analysis revealed a polymorphism in the *fgl2* gene in patients that are susceptible to SARS and severe periodontitis.^48^,^51^ Lastly, a recent study has demonstrated abundant levels of FGL2 protein in livers of patients with hepatocellular carcinoma.^52^ Collectively, these clinical data support the hypothesis that FGL2 might be involved in regulation of immunity.

The role of FGL2 in regulation of adaptive immune responses was first shown by Chan et al.,^47^ who studied the molecular and functional properties of the protein *in vitro*. A recombinant FGL2, which was generated in a baculovirus expression system, inhibited the proliferation of T cells in response to stimulation with anti-CD3/CD28 antibodies, Con A, and alloantigens. The inhibitory effect of FGL2 on T cells was mediated through suppression of DC maturation, characterized by the inhibition of nuclear factor kappa beta (NF-κB) translocation to the nucleus resulting in down-regulation of CD80 and major histocompatibility complex (MHC) class II molecules. The suppressive effects of FGL2 were abrogated by a specific antibody directed against the C-terminal domain of FGL2, strongly suggesting that the carboxyl FRED region accounts for the regulatory activity of the molecule. The recombinant protein also polarized allogeneic T cell responses towards a Th2 cytokine profile with increased production of IL-10 and IL-4, and decreased secretion of Th1 cytokines, including IFN-γ and IL-2.^47^ In another study, it was shown that the absence of FGL2 was associated with accelerated cellular rejection in a xenotransplant model.^46^

## FGL2 AS AN EFFECTOR OF REGULATORY T CELLS

A number of groups have recently reported that regulatory T cells have increased *fgl2* gene transcription as detected by microarray gene analysis. Herman et al. were the first to report increased transcripts of *fgl2* in Treg cells isolated from the pancreas of diabetic mice.^34^ Subsequent studies by Rudensky et al. have also detected high expression of *fgl2* in Treg cells isolated from wild-type *Foxp3^gfp^* mice and *IL-2*^−/−^ mice. Up-regulation of *fgl2* expression was observed in Treg cells from *IL-2*^−/−^ mice that were treated with recombinant IL-2 for 24 hours.^33^ Rudensky and colleagues further showed a positive correlation between *Foxp3* and *fgl2* expression; however, the expression of *fgl2* was not under the direct control of Foxp3.^53^

In addition to CD4^+^CD25^+^ Treg cells, other subsets of regulatory T cells have also been found to express high levels of *fgl2*. A recent report showed that CD8αα^+^ regulatory T cells in the intestine of mice over-express *fgl2*, which was suggested to play a role in the function of these cells.^54^ Our laboratory has detected high production of FGL2 protein and *fgl2* mRNA in both primary and clones of DN regulatory T cells. Interestingly, high levels of FGL2 were produced by a functional clone of DN T cells, but were undetectable in a non-functional DN T cell clone. Furthermore, increased expression of *fgl2* in primary DN T cells correlated with their suppressive activity *in vitro*. Finally, Anegon et al. found increased expression of *fgl2* in CD8^+^CD45RC^low^ regulatory T cells that mediate allograft tolerance in a rat transplantation model (personal communication). The role of FGL2 in these different types of regulatory T cells is currently being evaluated.

In agreement with previous studies, high levels of *fgl2* transcripts were detected in Treg cells by real-time polymerase chain reaction (PCR).^35^ In *fgl2*^−/−^ mice, an increased number and percentage of Treg cells were found with a greater expression of Foxp3 compared with *fgl2*^+/+^ Treg cells; however, the suppressive activity of *fgl2*^−/−^ Treg cells was significantly impaired.^35^ Furthermore, antibody to FGL2 completely inhibited the activity of *fgl2*^+/+^ Treg cells *in vitro*, strongly supporting the contention that expression of FGL2 accounts for the suppressive activity of Treg cells.^35^ Consistent with FGL2 contribution to Treg cell activity, targeted deletion of the gene led to an increase in immune reactivity of DC, T cells and B cells, and the development of autoimmune glomerulonephritis in aged *fgl2*^−/−^ mice.^35^

## THE ROLE OF FGL2 IN AN EXPERIMENTAL MODEL OF FULMINANT VIRAL HEPATITIS

**Figure 2.**
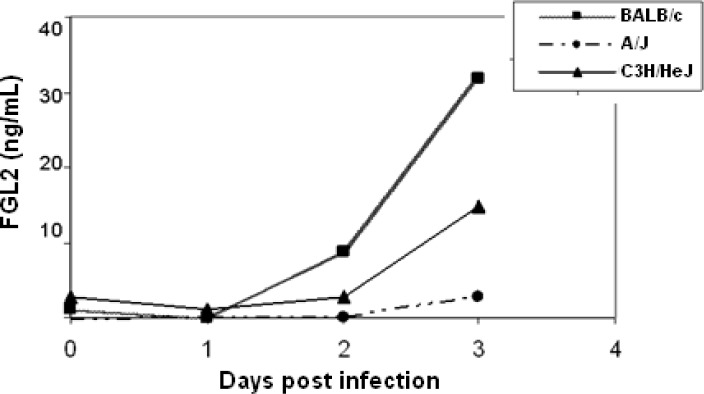
Levels of sFGL2 correlate with resistance and susceptibility in mouse strains following MHV-3 infection. Graph shows the levels of sFGL2 (ng/mL) in the plasma of different strains of mice following MHV-3 infection.

Our laboratory has extensively studied the role of FGL2 prothrombinase in a model of fulminant hepatitis caused by murine hepatitis virus strain 3 (MHV-3).^45^,^55^–^58^ Susceptible strains of mice develop a fatal hepatitis that is characterized by intravascular thrombosis and hepatocellular necrosis; resistant strains of mice survive and clear the virus within 10–14 days of infection. Several lines of evidence suggest that induction of FGL2 contributes to the lethality of MHV-3-induced hepatitis. First, only in susceptible BALB/cJ and semi-susceptible C3H/eJ mice is there induction of FGL2 by MHV-3.^56^,^59^,^60^ Of interest, BALB/cJ mice, which express high levels of FGL2, uniformly die of liver failure, whereas surviving C3H mice that express only moderate levels of FGL2 develop chronic hepatitis (Figure 2 and reference 36). In contrast, resistant A/J mice, which fail to produce FGL2 following MHV-3 infection either *in vivo* or *in vitro*, all survive and clear the virus (Figure 2 and reference 36). Second, treatment of susceptible mice with a monoclonal antibody to FGL2 prevents thrombosis, hepatic necrosis, and lethality of MHV-3 infection.^36^ Finally, targeted deletion of *fgl2* renders C57BL/6J mice largely resistant to MHV-3.^45^ At a molecular level, we have defined the active serine 89 site and requirement for phospholipids for prothrombinase activity of membrane-associated FGL2;^42^ demonstrated that *fgl2* is transcriptionally regulated by the nucleocapsid protein (N) of strains of MHV that cause lethal disease;^61^–^64^ and shown that the mechanism of lack of *fgl2* transcription in resistant A/J mice is altered phosphorylation of signal transducer and activation of transcription (STAT)1 α/β iso-forms.^64^

Recently we have developed an enzyme-linked immunosorbent assay (ELISA) to measure secreted FGL2 (sFGL2) in murine plasma.^36^ This assay was used to analyze plasma samples from both MHV-3 infected susceptible and resistant mice daily from the time of infection to day 8 post-infection.^36^ In our study, plasma levels of sFGL2 correlated with disease progression. MHV-3-susceptible mice expressed markedly elevated levels of sFGL2 over time, correlating with increased numbers of Treg cells, whereas resistant mice had no significant increase in levels of sFGL2 or Treg cells.^36^ Moreover, adoptive transfer of *fgl2*^+/+^ Treg cells into resistant *fgl2*^−/−^ mice increased their mortality following MHV-3 infection, demonstrating the importance of FGL2 as an effector of Treg cells in experimental viral hepatitis.^36^ These data collectively suggest that disturbances in Treg cell activity or number may be important in the pathogenesis of viral induced liver injury and that monitoring levels of sFGL2 may be of use in predicting outcome to infection.

## THE ROLE OF FGL2 IN THE PATHOGENESIS OF HCV INFECTION

**Figure 3.**
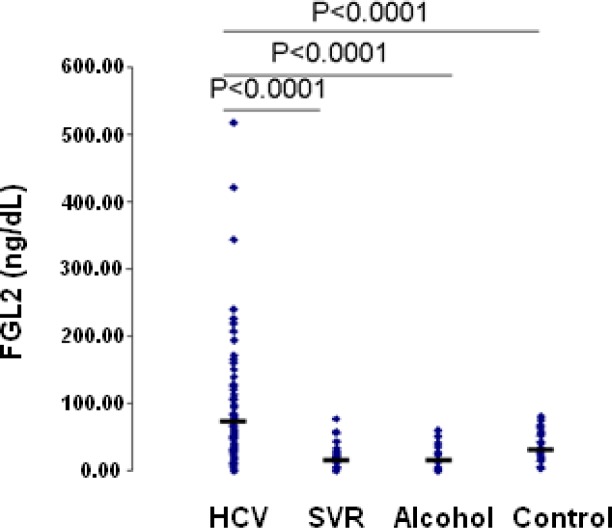
Mean plasma levels of sFGL2 in patients with chronic HCV infection. Ten (10) mL of heparinized blood was collected from 80 patients with chronic HCV infection, who had not received anti-viral therapy. Mean plasma levels of sFGL2 in these patients were compared to 30 healthy controls, 24 patients with inactive alcoholic cirrhosis, and 32 patients with chronic HCV who cleared the virus following successful anti-viral therapy (sustained virological responders (SVR)). Mean plasma levels of sFGL2 were significantly higher in patients with chronic HCV infection (84.3 ± 89.1 ng/mL, *n* = 80) compared to healthy controls (36.41 ± 21.9 ng/mL, *n* = 30), patients with alcoholic cirrhosis (18.8 ± 17.4 ng/mL, *n* = 24), or patients with SVR (16.6 ± 19.7 ng/mL, *n* = 32).

We have developed a sensitive and reproducible ELISA for measurement of sFGL2 in plasma samples in humans. We have established baseline levels of sFGL2 in healthy controls using plasma samples from healthy volunteers tested on two separate occasions. Plasma levels of sFGL2 were then compared to patients with chronic HCV infection as well as patients with non-viral related liver disease (alcohol-induced liver disease). Our data shown in Figure 3 suggest that, in patients with chronic HCV infection, levels of plasma sFGL2 is significantly higher than in patients with alcoholic liver disease, patients with a sustained viral response to anti-viral treatment, and healthy controls (Figure 3). These data demonstrate that in HCV patients, who cleared the virus following anti-viral therapy and developed an SVR, levels of sFGL2 return to levels seen in normal healthy controls. Furthermore, the results show that the presence of liver disease alone does not correlate with plasma levels of sFGL2 since patients with alcoholic cirrhosis have levels comparable to the healthy controls. Taken together, these preliminary results indicate that the level of sFGL2 may be a useful biomarker of disease progression and response to therapy in patients with HCV infection.

**Figure 4.**
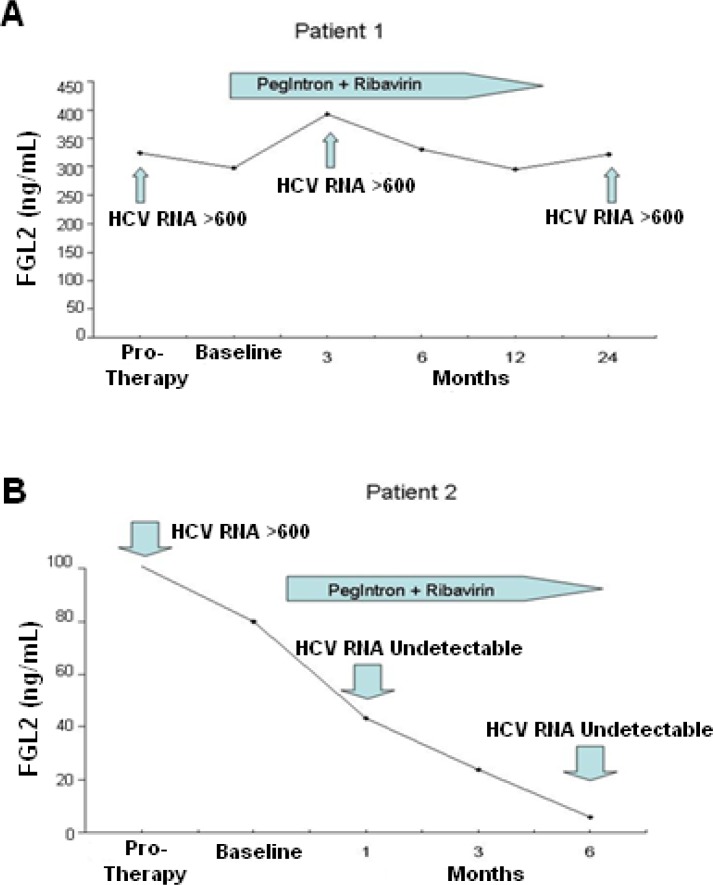
Time course of sFGL2 levels in two patients with chronic HCV infection treated with anti-viral therapy. A) Patient 1 with genotype 1 infection did not respond to 48 weeks of therapy with pegylated interferon and Ribavirin. Plasma sFGL2 levels in this patient were high prior to initiation of therapy, throughout treatment and 6 months after completion of therapy. B) Patient 2 with genotype 2 infection responded to anti-viral therapy. Plasma levels of sFGL2 were low prior to initiation of treatment and fell within 4 weeks of therapy to levels similar to healthy controls and remained very low after completion of therapy.

Preliminary data also demonstrated a significant difference in plasma levels of sFGL2 between HCV patients with genotype 1 compared to genotype 2/3 patients (120 versus 45 ng/mL). The data to date suggest that patients with high levels of plasma sFGL2 (>150 ng/mL) have a more vigorous form of HCV with a higher probability of being non-responders to anti-viral therapy, whereas patients with levels <100 ng/mL are more likely to respond to anti-viral treatment. This is demonstrated in Figure 4, which shows the time-course of sFGL2 levels in two representative patients with chronic HCV infection treated with anti-viral therapy. Patient 1 did not respond to 48 weeks of therapy with pegylated interferon and ribavirin. Plasma sFGL2 levels in patient 1 were very high prior to initiation of therapy, >300 ng/mL, and remained high throughout treatment and at 6 months post-treatment. In contrast, patient 2 had sFGL2 levels of less than 100 ng/mL prior to initiation of treatment; the level of sFGL2 fell within 4 weeks of therapy to levels seen in healthy controls, and levels of sFGL2 remained very low after completion of therapy.

**Figure 5.**
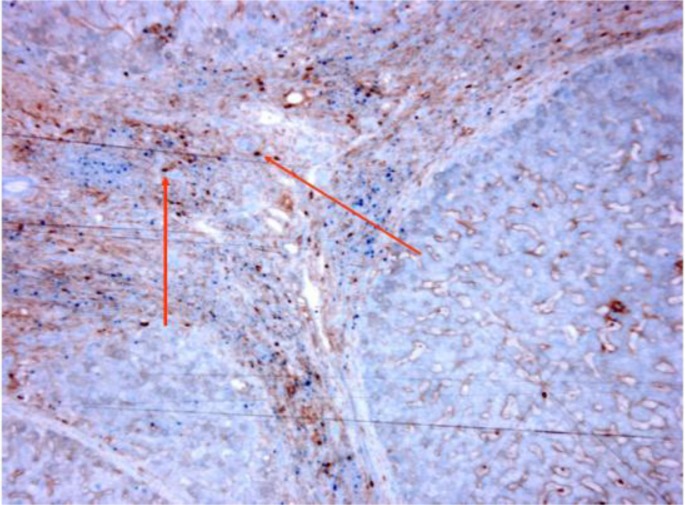
Pathological evidence for the interplay between Treg cells and FGL2 in patients with HCV infection. Figure shows immunohistochemistry staining of FGL2 (brown = membrane and cytoplasmic) and Foxp3 (blue = nuclear) in an explanted liver from an HCV patient. Some lymphocytes (arrows) co-express both FGL2 and Foxp3.

We now also have preliminary pathological evidence for the interplay between Treg cells and FGL2 in patients with HCV infection. Figure 5 shows the co-expression of FGL2 (membrane and cytoplasmic) and Foxp3 (nuclear), the master transcription factor of Treg cells, in some of the inflammatory cells in the liver of a patient with chronic HCV infection.

**Figure 6.**
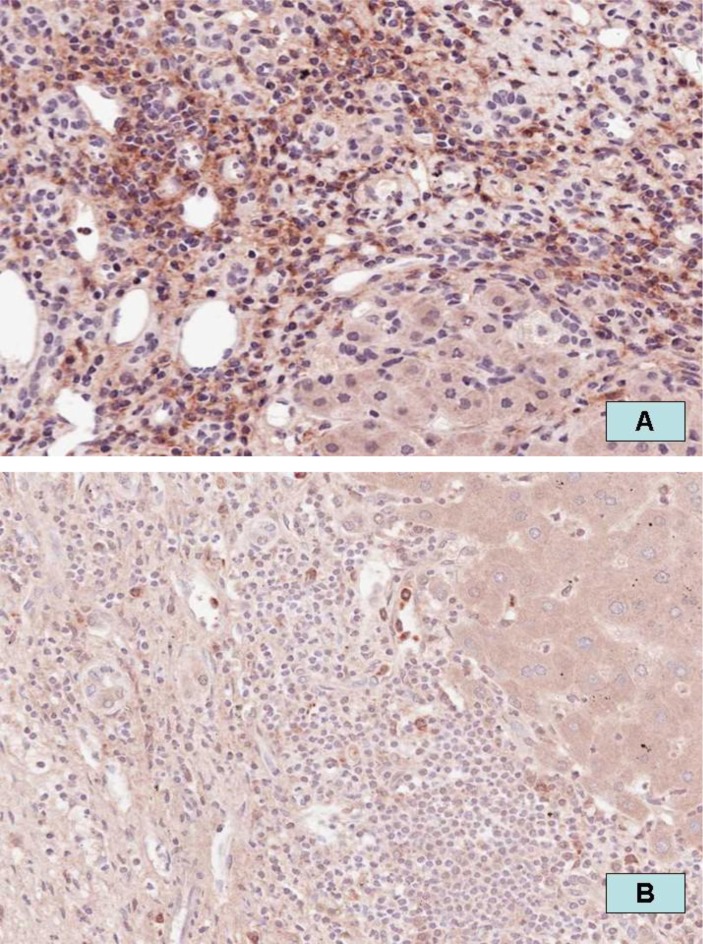
Increased expression of FGL2 in the explanted liver correlates with severity and recurrence of HCV infection. A: Chronic HCV patient with many FGL2-positive cells (brown cells) in the explant, who developed aggressive recurrent disease that did not respond to treatment. B: Chronic HCV patient who had only a few scattered FGL2-positive cells (brown cells) in the explant and has not required any treatment for recurrent HCV infection. Staining for FGL2 was performed by standard immunohistochemistry using a monoclonal anti-FGL2 antibody and the horse-radish immunoperoxidase method; magnification ×300.

In a preliminary study, we found that patients with high levels of FGL2 in the explanted liver are much more likely to have rapid and aggressive recurrence of HCV that responds poorly to treatment. Examples of the differences in the degree of FGL2 expression in the explanted liver of two patients and the correlation with the post-transplant clinical course are illustrated in Figure 6. Panel A shows a chronic HCV patient with many FGL2-positive cells (brown cells) in the explant who developed aggressive recurrent disease that did not respond to treatment. Panel B shows another chronic HCV patient who had only a few scattered FGL2-positive cells (brown cells) in the explanted liver and has not required any treatment for recurrent HCV infection.

## FGL2: MECHANISM OF ACTION

**Figure 7.**
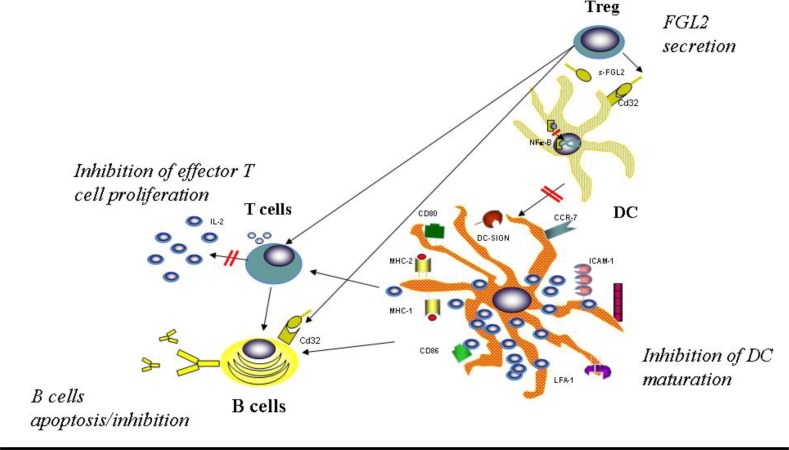
A proposed model of FGL2 immunoregulatory activities. Treg production of FGL2 down-regulates adaptive immune responses through binding to the inhibitory FcγRIIB receptor, which is expressed on antigen-presenting cells. The suppressive activities of FGL2 result in inhibition of the immune response against the HCV, leading to viral persistence and chronic infection. DC, dendritic cells.

Based upon the data collected to date, it has been shown that FGL2 is integral to both the innate and the adaptive immune responses. This is not surprising as FGL2 is a molecule that has been conserved through evolution from single-cell organisms such as the ameba to higher primates. We propose a mechanistic model by which FGL2 exerts its immunoregulatory effects (Figure 7). Treg cells secrete FGL2, which then binds to the inhibitory FcγRIIB receptor expressed on DC. Binding of FGL2 to FcγRIIB down-regulates immune activation of DC as indicated by inhibition of expression of the maturation markers CD80, CD86, and MHCII. This suppressive effect of FGL2 on DC was shown to be mediated through inhibition of NF-κB nuclear translocation.^47^ DC that are exposed to FGL2 would be therefore less effective in inducing proliferation and effector function of helper and cytotoxic T lymphocytes. Suppression of helper T cell activation and DC maturation by FGL2 could lead indirectly to inhibition of T-dependent and T-independent B cell responses, respectively. As demonstrated by our *in vitro* studies, FGL2 can also directly induce apoptosis in B cells upon binding to the inhibitory FcγRIIB receptor, which is known to be expressed on B cells. The indirect and direct suppressive activities of FGL2 result in inhibition of the immune response against the HCV, leading to viral persistence and chronic infection.

## CONCLUSIONS AND FUTURE DIRECTIONS

HCV infection is a major world health problem and the leading cause of HCC worldwide. Disturbances in Treg cell function or number have now been shown to contribute to failure of clearance of HCV and the development of chronic hepatitis. FGL2 has been shown to be an important effector molecule of Treg cells and was demonstrated to play a key role in the pathogenesis of both experimental and human viral hepatitis. Measurement of levels of sFGL2 in plasma of patients appears to predict both the course of HCV disease and response to anti-viral therapy, and, as such, FGL2 as a biomarker may become an important diagnostic reagent in the management of HCV patients. Furthermore, the studies show that inhibition of FGL2 improves outcomes to experimental hepatitis and thus provide impetus for generation of reagents to inhibit FGL2 in patients with acute and chronic hepatitis B and C virus infection either alone or in combination with present anti-viral agents. As expression of FGL2 has been shown to be associated with other diseases including HIV, SARS, and cancer, measurement of sFGL2 levels and development of reagents that interfere with FGL2 may have even broader applicability.

## Abbreviations:

DC: dendritic cells
FcγR: Fc gamma receptors
FGL2: fibrinogen-like protein 2
FRED: fibrinogen-related domain
HBV: hepatitis B virus
HCV: hepatitis C virus
IL: interleukin
MHC: major histocompatibility complex
MHV-3: murine hepatitis virus strain 3
SVR: sustained virological response
Treg cells: Foxp3^+^CD4^+^CD25^+^ regulatory T cells.
